# Bidirectional Association between Major Depressive Disorder and Gastroesophageal Reflux Disease: Mendelian Randomization Study

**DOI:** 10.3390/genes13112010

**Published:** 2022-11-02

**Authors:** Yuyang Miao, Shuai Yuan, Ye Li, Jie Chen, Xue Li, Susanna C. Larsson, Qiang Zhang

**Affiliations:** 1 Department of Geriatrics, Tianjin Medical University General Hospital, Tianjin 300052, China; 2 Tianjin Geriatrics Institute, Tianjin 300052, China; 3 Unit of Cardiovascular and Nutritional Epidemiology, Institute of Environmental Medicine, Karolinska Institutet, 17177 Stockholm, Sweden; 4 Centre for Global Health, Zhejiang University School of Medicine, Hangzhou 310030, China; 5 Department of Gastroenterology, The Third Xiangya Hospital, Central South University, Changsha 410017, China; 6 School of Public Health and the Second Affiliated Hospital, Zhejiang University School of Medicine, Hangzhou 310030, China; 7 Centre for Global Health, Usher Institute, The University of Edinburgh, Edinburgh EH8 9YL, UK; 8 Unit of Medical Epidemiology, Department of Surgical Sciences, Uppsala University, 75236 Uppsala, Sweden

**Keywords:** gastroesophageal reflux disease, major depressive disorder, Mendelian randomization analysis

## Abstract

Background: Observational research has found a bidirectional relationship between major depressive disorder and gastroesophageal reflux disease; however, the causal association of this relationship is undetermined. Aims: A bidirectional Mendelian randomization study was performed to explore the causal relationships between major depressive disorder and gastroesophageal reflux disease. Methods: For the instrumental variables of major depressive disorder and gastroesophageal reflux disease, 31 and 24 single-nucleotide polymorphisms without linkage disequilibrium (*r*^2^ ≤ 0.001) were selected from relevant genome-wide association studies, respectively, at the genome-wide significance level (*p* ≤ 5 × 10^−8^). We sorted summary-level genetic data for major depressive disorder, gastroesophageal reflux disease, gastroesophageal reflux disease without esophagitis, and reflux esophagitis from meta-analysis study of genome-wide association studies involving 173,005 individuals (59,851 cases and 113,154 non-cases), 385,276 individuals (80,265 cases and 305,011 non-cases), 463,010 individuals (4360 cases and 458,650 non-cases), and 383,916 individuals (12,567 cases and 371,349 non-cases), respectively. Results: Genetic liability to major depressive disorder was positively associated with gastroesophageal reflux disease and its subtypes. Per one-unit increase in log-transformed odds ratio of major depressive disorder, the odds ratio was 1.31 (95% confidence interval [CI], 1.19–1.43; *p* = 1.64 × 10^−8^) for gastroesophageal reflux disease, 1.51 (95% CI, 1.15–1.98; *p* = 0.003) for gastroesophageal reflux disease without esophagitis, and 1.21 (95% CI, 1.05–1.40; *p* = 0.010) for reflux esophagitis. Reverse-direction analysis suggested that genetic liability to gastroesophageal reflux disease was causally related to increasing risk of major depressive disorder. Per one-unit increase in log-transformed odds ratio of gastroesophageal reflux disease, the odds ratio of major depressive disorder was 1.28 (95% confidence interval, 1.11–1.47; *p* = 1.0 × 10^−3^). Conclusions: This Mendelian randomization study suggests a bidirectional causal relationship between major depressive disorder and gastroesophageal reflux disease.

## 1. Introduction

In recent years, major depressive disorder (MDD) has been recognized as an serious public mental health issue with an increasing disease burden [1], affecting approximately 264 million people worldwide [2]. Importantly, MDD has a high heritability of about 35%, and the age-of-onset of depression is not time-specific but throughout a person’s lifespan [3]. Gastroesophageal reflux disease (GERD) is believed to be a type of chronic disorder commonly existing in children and adults, which manifests common phenotypes including reflux esophagitis and gastroesophageal reflux disease without esophagitis [4]. It is estimated that around 20% of the population of Europe and the United States was affected by GERD by 2005 [5]. Bidirectional associations between MDD and GERD have been gradually found in observational studies [6,7,8,9], indicating that people with MDD may be at increased risk of GERD through unhealthy lifestyle and physiological dysfunction, such as collapse of the lower esophageal sphincter. On the other hand, GERD may increase MDD risk by affecting the central nervous system through chronic mucosal inflammation. However, the causality of the association between MDD and GERD is unknown, because of potential drawbacks in these studies, such as residual confounding and reverse causality in observational research.

Mendelian randomization (MR) is a statistic method of epidemiological studies using genetic variants as instrumental variables for the exposure, which contributes to evaluating the causal nature of the associations between exposure elements and outcome events [10]. The advantage of MR is that residual confounding can be minimized as the genetic variant is randomly allocated at conception and therefore is not affected by self-selected lifestyle and environmental factors [10]. Moreover, it can overcome reverse causality because genetic variants cannot be modified by disease status [10]. In this study, we conducted a two-sample MR analysis in order to elucidate the potential bidirectional relationship between MDD and GERD.

## 2. Materials and Methods

### 2.1. Study Design

**Figure 1:**
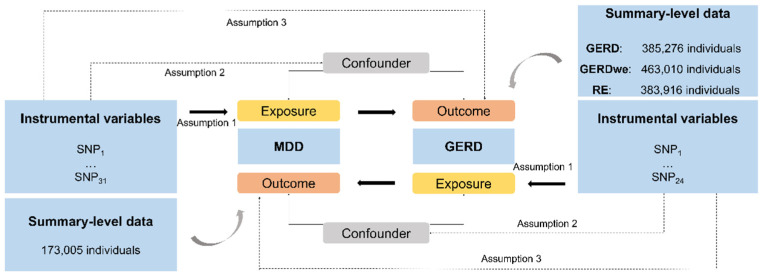
The present study design overview. All individuals are of European ancestry. MDD, Major Depressive Disorder; GERD, gastroesophageal reflux disease; SNP, single-nucleotide polymorphism.

Mendelian randomization is an epidemiological statistical analysis with a strength in causal inference based on instrumental variables (i.e., single nucleotide polymorphisms, SNPs) which are highly linked with the exposures (e.g., MDD) [11]. There are three important assumptions for MR analysis [11]. Firstly, the genetic variants used as instrumental variables ought to be highly related to the exposure elements. Secondly, the selected genetic variants cannot have associations with any confounders. Thirdly, the exposures should be the only pathway through which the genetic variants exert effects on the outcome [10]. Ethical approval was not required for this study, which was conducted on summary-level genetic data from publicly available databases of large-scale genome-wide association studies (GWASs). The overall scheme of the present study is shown in Figure 1.

### 2.2. Genetic Instrument and Data Sources for MDD

In a meta-analysis study of 7 large GWASs on MDD including 480,359 total participants (135,458 cases and 344,901 non-cases) of European ancestry, 44 SNPs associated with MDD were obtained at the genome-wide significance threshold (*p* ≤ 5 × 10^−8^) [12]. Linkage disequilibrium (LD) of these SNPs was estimated with the 1000 Genomes European reference panel. Then SNPs in LD (*r*^2^ > 0.001 or clump window < 10,000 kb) were discarded and the SNP with the lowest *p* values for the GWAS association was attained, leaving 29–31 SNPs as instrumental variables for MDD (Supplementary Tables S1–S3). Summary-level genetic data on MDD were selected in a GWAS meta-analysis study of the UK Biobank study, Psychiatric Genomics Consortium, deCODE genetics, Generation Scotland, Adult Health and Aging (GERA) Cohort, and iPSYCH, including 173,005 participants (59,851 cases and 113,154 non-cases) (Supplementary Table S4) (23 and Me was excluded).

### 2.3. Genetic Instrument and Data Sources for GERD

For genetic instrumental variables, we chose 25 SNPs robustly related to GERD with a genome-wide significance level *(p* ≤ 5 × 10^−8^) in a GWAS meta-analysis study of 5 studies involving 385,276 individuals (80,265 cases and 305,011 non-cases) [13]. After discarding SNPs in LD (*r*^2^ > 0.001 or clump window < 10,000 kb), 24 independent SNPs were used as genetic instrumental variables for GERD in the reverse-direction MR study (Supplementary Table S5). Summary-level data for GERD were collected through a GWAS meta-analysis of the UK Biobank database and QSkin cohorts, comprising 332,601 participants of European-descent (71,522 cases and 261,079 non-cases) [13] (23 and Me was excluded). Summary-level data for GERD without esophagitis and reflux esophagitis were available from the UK Biobank study, including 463,010 individuals (4360 cases and 458,650 non-cases) and 383,916 individuals (12,567 cases and 371,349 non-cases), respectively (Supplementary Table S6).

### 2.4. Statistical Analyses

We performed the random-effects model of the inverse-variance weighted (IVW) method as the principal statistical analysis [14]. Then we assessed whether the associations were consistent, as well as observed and corrected for potential pleiotropy through integrating 4 sensitivity analyses, including the weighted median [15], MR-Egger regression [16], MR Pleiotropy RESidual Sum and Outlier (MR-PRESSO) [17], and contamination mixture [18] methods. In the weighted median method, we tested whether at least half of the weights were provided by proper instrumental variables; if so, causal estimates were consistent [15]. MR-Egger regression was used to detect and correct for potential directional pleiotropy caused by violation of the third assumption of MR (i.e., the genetic variants have effects on the outcome not—or not completely—through the exposure of interest) [16]. The MR-PRESSO method is able to exclude observed genetic variant outliers and reassess the estimates after removing the outliers. The built-in distortion test will calculate the differences between original estimates and those after outlier removal [17]. The contamination mixture method is used to generate solid causal estimates from a load of genetic variants with the existence of invalid SNPs [18]. In order to measure the heterogeneity in these analyses, Cochrane’s Q value was calculated. The F-statistics value was calculated to evaluate the power of each analysis via online tools [19]. We then scaled the odds ratios (ORs) and confidence intervals (CIs) to 1-unit increase in log-transformed OR of MDD and GERD. All *p* values were 2-tailed and the analyses were performed using the TwoSampleMR [20], MR-PRESSO [17], and Mendelian Randomization [21] packages in R software (version 4.0.2; R Foundation for Statistical Computing, Vienna, Austria).

## 3. Results

### 3.1. Causal Effect of MDD on GERD

**Figure 2:**
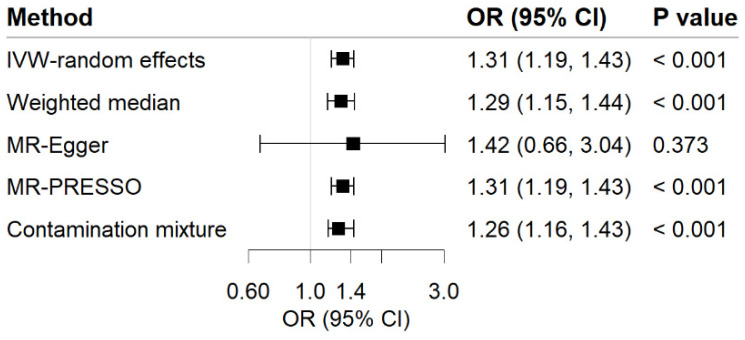
Associations of MDD with risk of GERD in MR analyses. The ORs of GERD were scaled to a 1-unit increase in log OR of MDD. IVW, inverse variance weighted; OR, odds ratio; CI, confidence interval; MR, Mendelian randomization.

**Figure 3:**
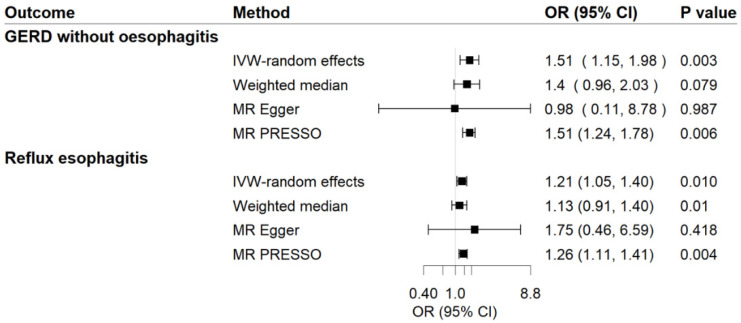
Associations of MDD with risk of GERD phenotypes in MR analyses. The ORs of GERD phenotypes were scaled to a 1-unit increase in log OR of MDD. IVW, inverse variance weighted; OR, odds ratio; CI, confidence interval; MR, Mendelian randomization.

Genetic predisposition to MDD has a positive causal effect on GERD risk (Figure 2). The OR of GERD per genetically predicted 1-unit increase in log-transformed OR of MDD was 1.31 (95% CI, 1.19–1.43; *p* = 1.64 × 10^−8^). For common phenotypes of GERD, the OR of GERD without esophagitis was 1.51 (95% CI, 1.15–1.981; *p* = 0.003) per 1-unit increase in log-transformed OR of MDD, besides, the OR of reflux esophagitis was 1.21 (95% CI, 1.05–1.40; *p* = 0.010) per 1-unit increase in log-transformed OR of MDD. The effects were validated as consistent in the following sensitivity analyses (Figure 2 and Figure 3). The F-statistic for MDD instruments was 530.47, and the power was 84%, suggesting the strong power of the analyses. Although there was moderate heterogeneity for GERD, GERD without esophagitis, and reflux esophagitis (Cochrane’s Q = 53.54, 32.21, and 23.56, respectively) were observed, no indication of horizontal pleiotropy was found through the intercept test in MR-Egger method (Intercept = −0.003, *p* for intercept = 0.829; Intercept = 0.014, *p* for intercept = 0.702; Intercept = −0.012, *p* for intercept = 0.590) and no genetic variant outliers were detected by MR-PRESSO method.

### 3.2. Causal Effect of GERD on MDD

**Figure 4:**
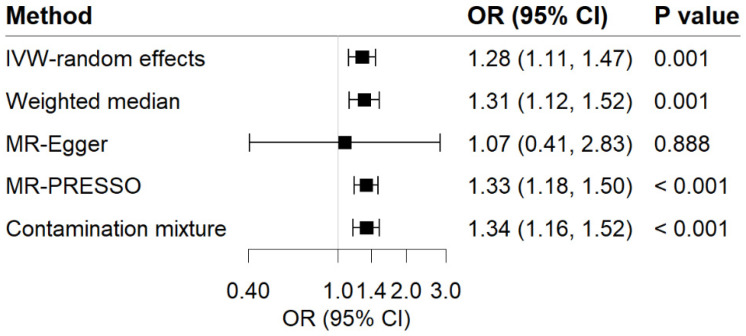
Associations of GERD with risk of MDD in MR analyses. The ORs of MDD were scaled to a 1-unit increase in log OR of GERD. IVW, inverse variance weighted; OR, odds ratio; CI, confidence interval; MR, Mendelian randomization.

For the reverse-direction MR study, genetic liability to GERD displayed a positive causal relationship with MDD risk (Figure 4). Per 1-unit increase in log-transformed OR of GERD showed a causal effect on MDD (OR = 1.28; 95% CI, 1.11–1.47; *p* = 1.0 × 10^−3^). Sensitivity analyses observed a consistent association (Figure 4). We noticed the heterogeneity in this analysis (Cochrane’s Q = 47.06) but no evidence supporting directional pleiotropy (Intercept = 0.006; *p* for intercept = 0.725). MR-PRESSO analysis detected 1 outlier; however, the causal association remained persistent after removing this outlier. The F-statistic for GERD genetic instruments was calculated as 393.35, and the power was 56%, showing the relatively strong power of the analyses. 

## 4. Discussion

In our MR analysis, we uncovered bi-directional positive causal relationships between MDD and GERD. In the forward-direction MR study, genetically predicted MDD was positively related with risk of GERD, the risk of GERD without esophagitis and reflux esophagitis, which supports findings from most observational studies [7,9,22]. A cross-sectional study including 4790 MDD patients and 728,749 patients with GERD found that having depression diagnosis significantly increased the risk of GERD (OR, 3.16, 95% CI, 2.71–3.68) [22]. A prospective cohort study with 84,873 participants followed-up for 3.3 years also found that the hazard ratio of GERD was 1.72 (95% CI, 1.60–1.85) in MDD patients compared with the non-patients [7]. Interestingly, patients treated with tricyclic antidepressant after diagnosis of depression had an increased risk of GERD, while use of serotonin reuptake inhibitors (another type of antidepressant) was not associated with GERD [7]. 

The reverse-direction MR analysis observed a positive effect of GERD on MDD, which is in line with other studies [6,8,9,23,24,25]. An observational study with 1612 community-based Australian found that GERD was independently associated with MDD with an OR of 2.6 (95% CI, 1.7–3.8) after accounting for confounder [23]. A case-control study including 65,333 participants reported a 1.7-fold increased risk of reflux in depressed individuals [24]. A longitudinal perspective cohort study involving 3813 GERD patients and 15,252 matched controls without GERD revealed that GERD tripled the risk of subsequent depressive disease [8]. Furthermore, another research of two nested case-control studies (60,957 depressed patients and 243,828 controls in study I, and 133,089 GERD patients and 266,178 controls in study II) found a bidirectional association between GERD and depression [25]. Our study strengthened the association and implied this association is highly likely to be causal, using MR analysis.

There are various corresponding mechanisms supporting the bidirectional relationship between MDD and GERD. The esophageal mucosa of GERD patients has high levels of cytokines and chemokines, which activate the recruitment and migration of immune cells [26,27]. This chronic peripheral inflammation may lead to upregulation of inflammatory response in the central nervous system, which exerts a critical pathophysiological effect in the subsequent progression of depression [28,29,30]. Moreover, when GERD occurs, acid reflux activates the autonomic nervous system and increases vagus nerve activation which causes bronchial constriction, leading to sleep disorders and mood disorders [25,31,32]. The risk of GERD may be increased by depression through reducing pressure on the lower esophageal sphincter and increasing gastric acid secretion [33]. Besides, it can also lower the threshold of sensation and increase sensitivity to the esophageal stimulation [33]. Antidepressant use may be another possible factor exacerbating reflux [34].

There are a list of strengths and limitations in our study. In observational studies, the results are easily affected by reverse causality as well as confounding. The main advantage of this research is MR analysis design, which diminished these limitations. Another strength is that all included participants were of European ancestry, which reduced population stratification bias; however, the generalizability of our findings to populations of different descents might be limited by the population confinement. Furthermore, the calculated F statistics and power for MDD and GERD show relatively strong power for the analyses. Moreover, the causally positive associations between MDD and GERD phenotypes are investigated in the study, which provides potential causalities between MDD and GERD subtypes; however, due to the lack of adequate instrument variables for GERD subtypes, the reverse causal associations between GERD subtypes and MDD still need to be explored in the future. When interpreting the MR studies, there are various limitations. Pleiotropy was one major issue. However, no suggestion of horizontal pleiotropy was detected in direction MR-Egger analyses and the associations remained stable in MR-PRESSO analyses, which indicated that pleiotropy bias should be minimal. In addition, there was 10.5% of sample overlap between exposure element and outcome event data, which might result in overfitting of the model and make the causal estimates tend towards observational associations. However, our analyses were based on data from GWAS meta-analyses including a large number of cases and controls. Thus, this bias caused by a small population overlap might not be an important issue in the causal inference. Besides, the information regarding detailed LA classification of reflux esophagitis is not available from the present GWAS study, which limits us to exploring the causal effect between MDD and the progression of reflux esophagitis.

In conclusion, in this MR study, we found a bi-directional causal relationship between MDD and GERD. These findings suggest paying attention to MDD prevention in patients with GERD as well as GERD prevention in individuals with high risk of MDD.

## Data Availability

Ethical approval was not required for this study which was conducted on summary-level genetic data from publicly available databases of large-scale genome-wide association studies.

## Supplementary Materials

The following supporting information can be downloaded at: https://www.mdpi.com/article/10.3390/genes13112010/s1, Table S1: Selected instrumental variables for major depressive disease and their associations with gastroesophageal reflux disease. Table S2: Selected instrumental variables for major depressive disease and their associations with gastroesophageal reflux disease without esophagitis. Table S3: Selected instrumental variables for major depressive disease and their associations with reflux esophagitis. Table S4: Detailed information on used studies for major depressive disease. Table S5: Selected instrumental variables for gastroesophageal reflux disease and their associations with major depressive disease. Table S6: Detailed information on used studies for gastroesophageal reflux disease.
